# Ovarian Cancer and High Body‐Mass Index: A Global Burden of Disease Database Study From 1990 to 2021

**DOI:** 10.1002/cam4.71541

**Published:** 2026-02-05

**Authors:** Xiaolong Li, Guangming Fu, Zhongyu Liu, Jiwei Li, Dongyong Shan

**Affiliations:** ^1^ Department of Oncology The Second Xiangya Hospital, Central South University Changsha China

**Keywords:** BMI, epidemiology, GBD, obesity, ovarian cancer

## Abstract

**Background:**

Excess adiposity has been recognized as a significant modifiable risk factor for ovarian cancer (OC), but the epidemiology of OC attributable to high BMI remain largely unknown.

**Methods:**

Utilizing comprehensive data from the Global Burden of Disease 2021 study, this investigation quantifies the epidemiological impact of elevated body mass index (BMI) on OC.

**Results:**

Our study demonstrated a striking 17,344 mortality cases attributable to BMI in 2021, with a 153.2% increase compared to 1990. The age‐standardized mortality rate (ASMR) and disability‐adjusted life years (DALYs) rate associated with excessive BMI rose from 0.18 per 100,000 (95% UI: −0.04–0.33) and 4.57 per 100,000 (95% UI: 0.94–8.6) in 1990 to 0.2 per 100,000 (95% UI: 0.05–0.36) and 5.46 per 100,000 (95% UI: 1.3–9.62) in 2021, respectively, with DALYs showing a 152.6% increase during this period. Notably, geriatric populations and low‐income nations exhibited disproportionately elevated mortality and DALY counts in 2021. The Bayesian age‐period‐cohort (BAPC) predictive modeling framework was used to quantify the average yearly rate of the obesity‐related OC and demonstrated a continued rise in both incidence and mortality rates over the next 25‐year period.

**Conclusion:**

These findings highlight metabolic dysfunction as a critical public health challenge in OC pathogenesis, emphasizing the urgent need to address modifiable metabolic determinants and associated conditions.

## 1 Introduction

Ovarian cancer (OC), a highly heterogeneous malignancy, primarily originates from the fimbriated end of the fallopian tube. It is responsible for approximately 4.7% of global cancer‐related deaths, contributing to an escalating health burden worldwide [1]. Recent epidemiological surveillance data reveals a progressive increase in ovarian cancer (OC) incidence, accompanied by significant geographic heterogeneity in disease distribution [2, 3]. The etiological pathways underlying OC pathogenesis remain incompletely understood, with a substantial proportion of patients experiencing suboptimal clinical outcomes despite advances in therapeutic modalities.

Prolactin (PRL) has been implicated in ovarian tumorigenesis through its metabolic and immunomodulatory effects and experimental evidence demonstrates that prolactin promotes fat accumulation [4, 5]. In vitro studies demonstrated that PRL promotes ovarian cancer cell proliferation and migration via activation of the JAK/STAT pathway, while clinical data show that high expression of PRL receptors (PRLR) in epithelial OC correlates with reduced survival [6]. Additionally, excessive adiposity has been shown to amplify both susceptibility to ovarian malignancies and adverse clinical outcomes, highlighting elevated BMI as a critical modifiable risk factor in ovarian cancer pathogenesis [7, 8, 9].

Utilizing the Global Burden of Disease 2021 (GBD 2021) dataset, this study quantifies the epidemiological impact of BMI‐driven ovarian cancer burden and projects disease trajectories over the next 25 years. The findings aim to inform evidence‐based policy formulation and optimize healthcare resource allocation.

## 2 Materials and Methods

### 2.1 Data Source

The datasets employed in this study were obtained from the Global Health Data Exchange (GHDx; https://ghdx.healthdata.org), a comprehensive repository for the Global Burden of Disease 2021 (GBD 2021) study. Ovarian cancer (OC) cases were systematically classified using the International Classification of Diseases‐10 (ICD‐10) code C56. Key metrics for assessing the disease burden included incidence rates, age‐standardized incidence rate (ASIR), disability‐adjusted life years (DALYs), age‐standardized mortality rate (ASMR), age‐standardized DALY rate (ASDR), and Bayesian age‐period‐cohort (BAPC) modeling framework. The GBD 2021 methodology framework, which incorporates updated epidemiological modeling techniques and global surveillance data, has been documented in prior studies [10, 11, 12].

The Socio‐demographic Index (SDI), a composite measure integrating per capita income and educational attainment levels, was stratified into five tiers to evaluate regional socioeconomic disparities: low (< 0.46), low‐middle (0.46–0.60), middle (0.61–0.69), high‐middle (0.70–0.81), and high (> 0.81). Projections of ASDR and age‐standardized incidence rate (ASIR) for ovarian cancer across various age cohorts were modeled for the period from 2022 to 2046. This study adhered to the ethical principles outlined in the Declaration of Helsinki and received approval from the Institutional Review Board of the Second Xiangya Hospital, Central South University, Changsha, China (ethical approval number: LYF20230131).

### 2.2 Disease Prediction

The Bayesian age‐period‐cohort (BAPC) modeling framework was applied to forecast age‐standardized mortality (ASMR) and age‐standardized DALY rate (ASDR) trends for OC. Model inputs included age groups (5‐year intervals), calendar years (1990–2021), and birth cohorts (stratified by 5‐year intervals of birth years). The Bayesian age‐period‐cohort (BAPC) model incorporated hierarchical Poisson regression with random walk priors to account for age‐specific, period‐specific, and cohort‐specific effects, as detailed in previous applications [10, 11, 12]. Nested Laplace approximation algorithms were used for parameter estimation, ensuring computational efficiency.

External validation was performed by splitting the dataset into training (1990–2016) and testing subsets (2017–2021). Model performance was evaluated using mean absolute error (MAE) and root mean square error (RMSE) against observed GBD 2021 data. The Bayesian age‐period‐cohort (BAPC) framework demonstrated superior accuracy compared to conventional generalized additive models (GAMs) and Poisson regression, particularly in capturing nonlinear temporal trends. Projections for 2022–2046 were generated using the validated model parameters.

## 3 Results

### 3.1 The Global Incidence and Mortality of Ovarian Cancer Attributable to BMI

**FIGURE 1:**
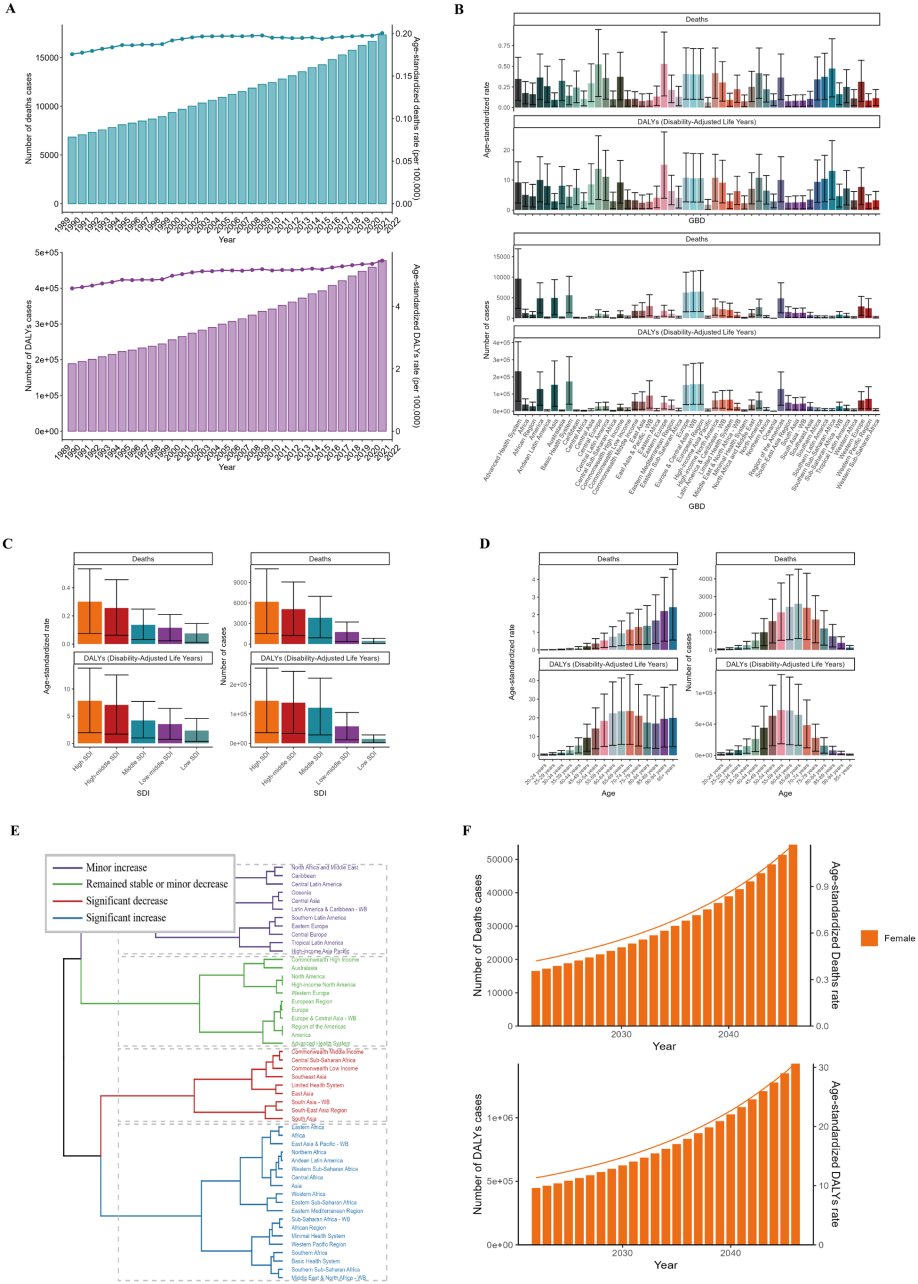
ASMR and ASDR of OC. (A) Numbers and age‐standardized rates of obesity‐related deaths and DALYs from 1990 to 2021. (B–E) Numbers and age‐standardized rates of obesity‐related deaths and DALYs in 2021 for different regions (B), SDI region (C), different age groups (D) and different regions (E) in 2021. (F) The predicted results in obesity‐related numbers and age‐standardized rates of deaths and DALYs globally from 1990 to 2046.

Since 1990, the global burden of ovarian cancer, as measured by mortality and disability‐adjusted life years (DALYs), has shown a consistent upward trend (Figures 1A and 3A). Specifically, deaths attributable to high BMI in ovarian cancer surged from 6850 cases (95% UI: 1423–12,865) in 1990 to 17,344 cases (95% UI: 4141–30,810) in 2021 (Figure 1A and Table 1). Similarly, BMI‐related DALYs for ovarian cancer rose sharply from 188,874 (95% UI: 38,401–355,691) in 1990 to 477,248 (95% UI: 113,449–840,002) in 2021 (Figure 1A and Table 2). Among the 50 Global Burden of Disease (GBD) regions, Europe recorded the highest obesity‐associated mortality (6276 deaths; 95% UI: 1558–11,230) and DALYs (152,187; 95% UI: 38,435–269,834) for ovarian cancer in 2021 (Figure 1B).

**TABLE 1:** The number of deaths cases and the age‐standardized deaths rate attributable to high BMI in 1990 and 2021, and its trends from 1990 to 2021 globally in ovarian cancer.

Characteristics	1990		2021		1990–2021
	Number of deaths cases (95% UI)	The age‐standardized deaths rate/100000 (95% UI)	Number of deaths cases (95% UI)	The age‐standardized deaths rate/100000 (95% UI)	EAPC (95% CI)
Global	6850 (1423–12,865)	0.18 (0.04–0.33)	17,344 (4141–30,810)	0.2 (0.05–0.36)	0.31 (0.24–0.39)
*Age*					
20–24 years	6 (−3–16)	0 (0–0)	31 (4–59)	0.01 (0–0.01)	4.51 (4.30–4.73)
25–29 years	19 (0–40)	0 (0–0.01)	67 (12–128)	0.01 (0–0.02)	3.46 (3.30–3.63)
30–34 years	45 (5–90)	0.01 (0–0.02)	138 (29–253)	0.02 (0–0.04)	2.37 (2.25–2.49)
35–39 years	96 (14–186)	0.03 (0–0.05)	264 (60–477)	0.05 (0.01–0.09)	1.66 (1.51–1.81)
40–44 years	202 (35–388)	0.07 (0.01–0.14)	527 (122–944)	0.11 (0.02–0.19)	1.04 (0.90–1.18)
45–49 years	361 (69–676)	0.16 (0.03–0.29)	995 (234–1767)	0.21 (0.05–0.37)	0.70 (0.54–0.85)
50–54 years	654 (129–1227)	0.31 (0.06–0.58)	1622 (390–2857)	0.36 (0.09–0.64)	0.54 (0.44–0.64)
55–59 years	819 (171–1534)	0.44 (0.09–0.83)	2111 (539–3772)	0.53 (0.14–0.95)	0.59 (0.51–0.68)
60–64 years	1073 (230–1980)	0.67 (0.14–1.23)	2424 (572–4224)	0.76 (0.18–1.32)	0.40 (0.31–0.49)
65–69 years	1121 (237–2115)	0.91 (0.19–1.71)	2596 (639–4547)	0.94 (0.23–1.65)	0.04 (−0.04–0.12)
70–74 years	865 (178–1621)	1.02 (0.21–1.91)	2367 (582–4316)	1.15 (0.28–2.1)	0 (−0.13–0.12)
75–79 years	795 (168–1494)	1.29 (0.27–2.43)	1711 (400–3050)	1.30 (0.30–2.31)	−0.01 (−0.17–0.16)
80–84 years	450 (84–857)	1.27 (0.24–2.42)	1197 (268–2208)	1.37 (0.31–2.52)	−0.01 (−0.23–0.21)
85–89 years	237 (45–451)	1.57 (0.30–2.98)	766 (167–1433)	1.68 (0.36–3.13)	0.21 (−0.05–0.48)
90–94 years	85 (17–164)	1.99 (0.40–3.84)	395 (89–738)	2.21 (0.50–4.13)	0.34 (0.12–0.55)
95+ years	21 (4–40)	2.04 (0.42–3.93)	133 (30–250)	2.43 (0.56–4.6)	0.43 (0.30–0.55)
*SDI region*					
High‐middle SDI	2244 (484–4207)	0.22 (0.05–0.42)	5095 (1241–9060)	0.26 (0.06–0.46)	0.38 (0.28–0.49)
High SDI	3802 (812–7120)	0.35 (0.07–0.65)	6187 (1531–10,979)	0.30 (0.08–0.53)	−0.56 (−0.69 to −0.43)
Low‐middle SDI	187 (13–392)	0.03 (0–0.06)	1771 (357–3220)	0.12 (0.02–0.21)	4.86 (4.66–5.06)
Low SDI	62 (0–139)	0.02 (0–0.05)	433 (68–840)	0.08 (0.01–0.15)	3.74 (3.65–3.83)
Middle SDI	543 (65–1064)	0.05 (0.01–0.10)	3833 (904–6972)	0.14 (0.03–0.25)	3.23 (3.15–3.30)

**TABLE 2:** The number of DALYs cases and the age‐standardized DALYs rate attributable to high BMI in 1990 and 2021, and its trends from 1990 to 2021 globally in ovarian cancer.

Characteristics	1990		2021		1990–2021
	Number of DALYs cases (95% UI)	The age‐standardized DALYs rate/100000 (95% UI)	Number of DALYs cases (95% UI)	The age‐standardized DALYs rate/100000 (95% UI)	EAPC (95% CI)
Global	188,874 (38401–355,691)	4.57 (0.94–8.6)	477,248 (113449–840,002)	5.46 (1.3–9.62)	0.47 (0.42–0.52)
*Age*					
20–24 years	428 (−213–1142)	0.09 (−0.04–0.23)	2182 (291–4194)	0.37 (0.05–0.7)	4.52 (4.30–4.74)
25–29 years	1227 (15–2643)	0.28 (0–0.6)	4413 (818–8377)	0.75 (0.14–1.42)	3.48 (3.31–3.64)
30–34 years	2714 (278–5417)	0.70 (0.07–1.41)	8328 (1763–15,166)	1.38 (0.29–2.51)	2.39 (2.27–2.51)
35–39 years	5255 (780–10,199)	1.49 (0.22–2.90)	14,528 (3287–26,276)	2.59 (0.59–4.68)	1.67 (1.52–1.82)
40–44 years	10,048 (1760–19,196)	3.51 (0.61–6.70)	26,242 (6096–46,794)	5.25 (1.22–9.35)	1.05 (0.91–1.20)
45–49 years	15,974 (3026–29,871)	6.88 (1.30–12.86)	44,223 (10412–78,821)	9.34 (2.2–16.65)	0.70 (0.55–0.86)
50–54 years	25,631 (5060–48,016)	12.06 (2.38–22.59)	63,725 (15272–112,607)	14.32 (3.43–25.31)	0.55 (0.45–0.65)
55–59 years	28,101 (5861–52,651)	15.17 (3.16–28.43)	72,665 (18503–129,616)	18.36 (4.68–32.75)	0.60 (0.51–0.68)
60–64 years	31,707 (6764–58,435)	19.74 (4.21–36.38)	71,900 (16919–125,493)	22.47 (5.29–39.21)	0.41 (0.33–0.50)
65–69 years	27,978 (5923–52,911)	22.63 (4.79–42.80)	64,891 (15980–114,009)	23.52 (5.79–41.33)	0.05 (−0.03–0.13)
70–74 years	17,738 (3662–33,308)	20.95 (4.33–39.34)	48,557 (11920–88,813)	23.59 (5.79–43.15)	−0.01 (−0.13–0.12)
75–79 years	12,958 (2735–24,415)	21.05 (4.44–39.66)	27,934 (6519–49,974)	21.18 (4.94–37.89)	−0.01 (−0.18–0.15)
80–84 years	5787 (1077–11,018)	16.36 (3.05–31.15)	15,338 (3436–28,286)	17.51 (3.92–32.3)	−0.03 (−0.25–0.2)
85–89 years	2406 (454–4574)	15.92 (3.00–30.27)	7757 (1686–14,497)	16.97 (3.69–31.71)	0.20 (−0.06–0.46)
90–94 years	750 (150–1452)	17.51 (3.49–33.88)	3477 (783–6504)	19.44 (4.38–36.36)	0.34 (0.12–0.55)
95+ years	172 (35–331)	16.87 (3.45–32.51)	1089 (249–2052)	19.97 (4.57–37.66)	0.39 (0.27–0.52)
*SDI region*					
High‐middle SDI	65,389 (13956–121,986)	6.28 (1.34–11.72)	138,126 (33461–244,871)	7.08 (1.71–12.59)	0.26 (0.18–0.35)
High SDI	96,742 (20587–180,828)	9.06 (1.93–16.94)	144,449 (36080–255,583)	7.81 (1.95–13.83)	−0.58 (−0.71 to −0.46)
Low‐middle SDI	6283 (459–13,117)	0.87 (0.06–1.83)	57,906 (11973–104,671)	3.56 (0.73–6.43)	4.84 (4.64–5.05)
Low SDI	2127 (24–4781)	0.78 (0.01–1.74)	14,943 (2349–28,748)	2.37 (0.37–4.57)	3.66 (3.57–3.76)
Middle SDI	17,976 (2083–35,888)	1.49 (0.18–2.94)	121,139 (28727–221,614)	4.22 (1–7.71)	3.28 (3.22–3.35)

**FIGURE 2:**
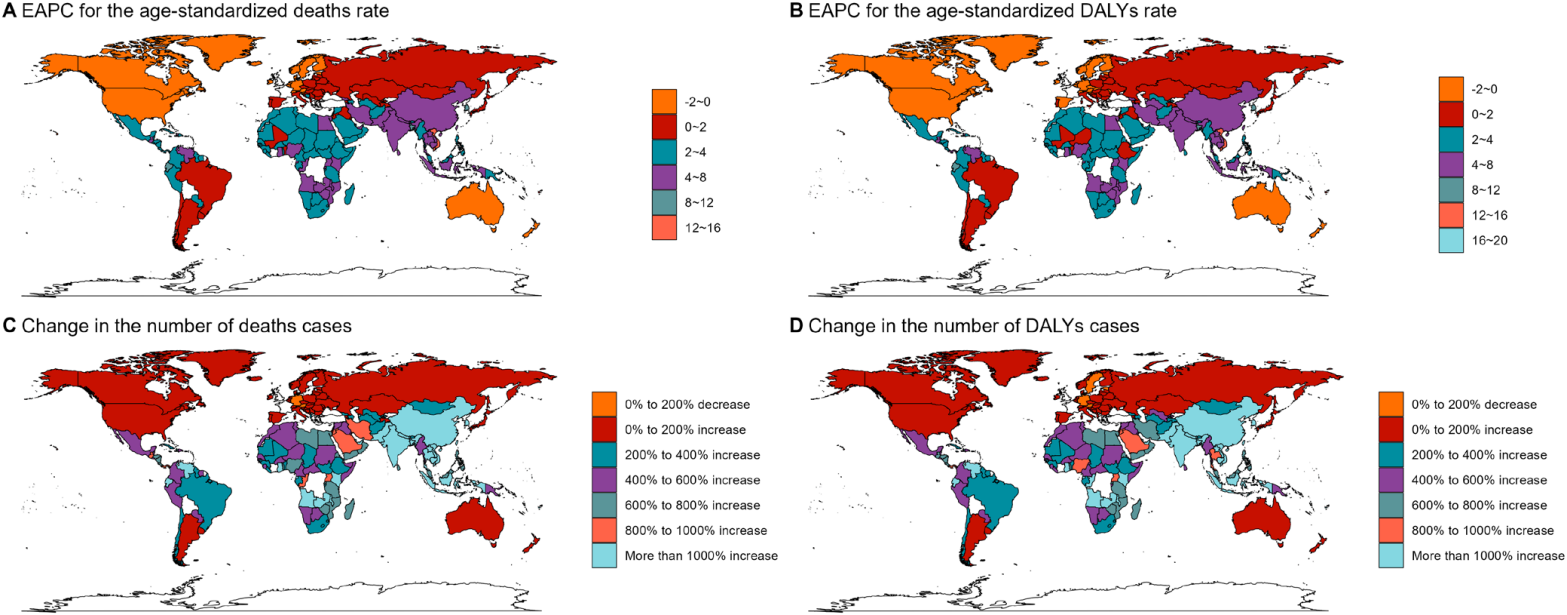
The ASMR, ASDR, number of death cases, and DALYs cases for OC in different regions and countries around the world.

In 2021, regions classified under a high socio‐demographic index (SDI) exhibited the highest ovarian cancer‐related mortality (3802 deaths) and DALYs (96,742 cases) (Figures 1C and 2). Notably, regions such as Africa reported significant increases in mortality and DALYs, while South Asia and other areas experienced substantial reductions (Figure 1E).

### 3.2 The Contribution of Elevated BMI to the Ovarian Cancer Disease Burden

The age‐standardized mortality rate (ASMR) and age‐standardized disability rate (ASDR) for ovarian cancer linked to elevated BMI increased from 0.18 (95% UI: −0.04–0.33) and 4.57 (95% UI: 0.94–8.6) per 100,000 in 1990 to 0.20 (95% UI: 0.05–0.36) and 5.46 (95% UI: −1.3–9.62) per 100,000 in 2021, respectively (Figure 1A; Tables 1 and 2).

**FIGURE 3:**
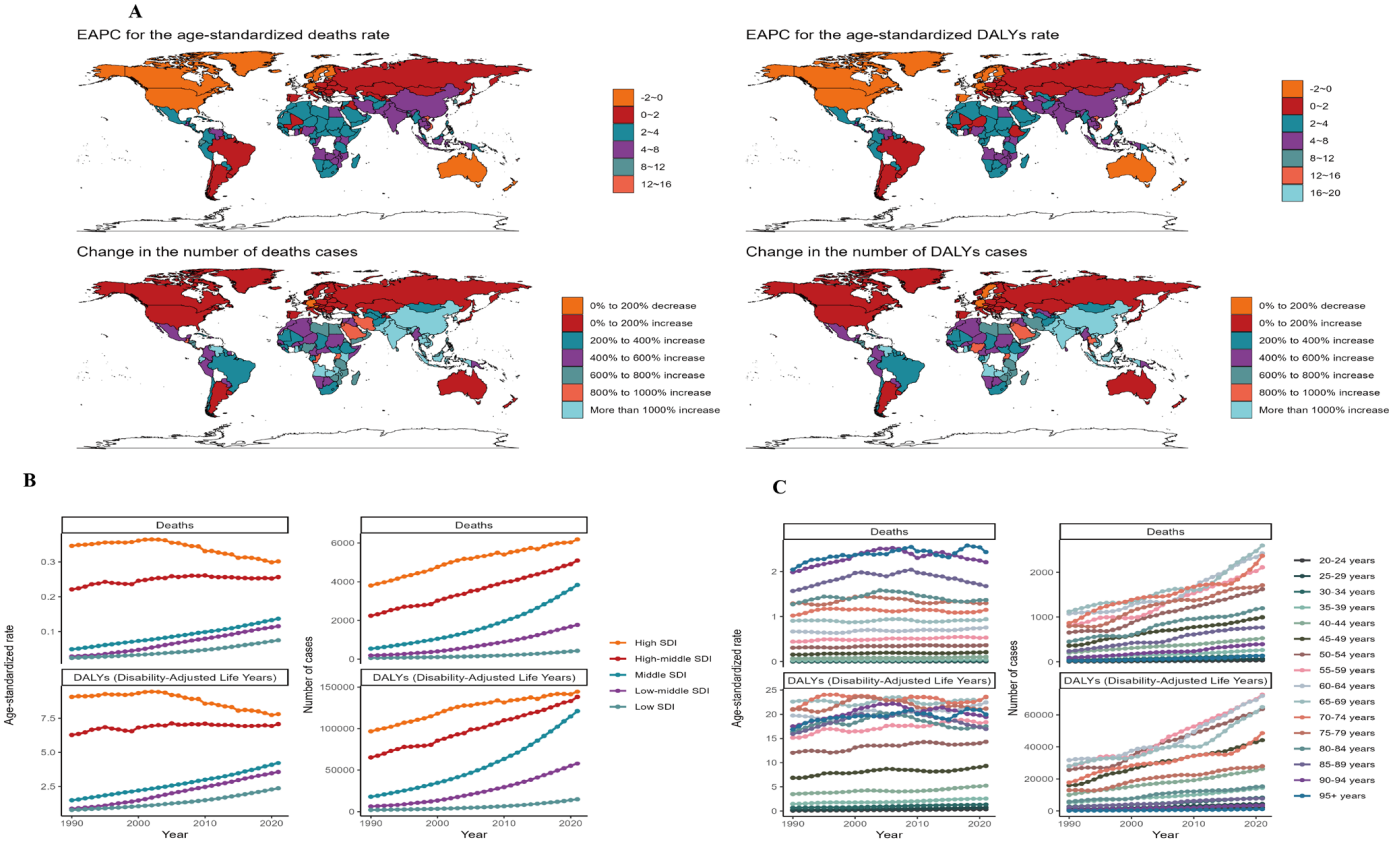
Change of the ASMR and ASDR of OC from 1990 to 2021. (A) EAPC for the age‐standardized rates of obesity‐related deaths and DALYs in 1990 and 2021. (B, C) The trend of the numbers and age‐standardized rates of obesity‐related deaths and DALYs from 1990 to 2021 for SDI region (B) and different age groups (C).

Age‐stratified analyses for 2021 revealed that BMI‐associated ASMR and ASDR increased with advancing age, peaking most prominently in individuals over 65 years old. Notably, ASDR exhibited fluctuations beyond age 74 (Figure 1D). The number of deaths and DALY cases followed a unimodal distribution, reaching maxima in the 55–59 and 65–69 age cohorts (Figure 1D). From 1990 to 2021, progressive increases in ASMR and ASDR were observed universally across all age cohorts and SDI regions (Figure 3B,C).

### 3.3 Predicting the Incidence and Mortality of Ovarian Cancer Associated With Elevated BMI

A goodness‐of‐fit‐optimized backpropagation (BP) neural network model was employed to project trends in ovarian cancer incidence and mortality linked to elevated BMI from 2022 to 2046. The model forecasts a continued rise in both incidence and mortality rates over the next 25‐year period. Projected estimates indicate 54,411 deaths and 1,426,513 disability‐adjusted life years (DALYs) attributable to BMI‐related ovarian cancer (Figure 1F).

## 4 Discussion

Over the past three decades, ovarian cancer (OC) has exhibited a significant global increase in incidence, mortality rates, and disability‐adjusted life years (DALYs). Elevated body mass index (BMI), a critical indicator of obesity, is widely recognized as a contributing factor to metastatic progression and poor clinical outcomes in this malignancy [13, 14, 15, 16]. Our analysis confirms that BMI‐related OC burden rose sharply from 1990 to 2021, with projections indicating continued escalation over the next 25 years. These findings underscore the urgent need to address metabolic risk factors in public health strategies targeting OC prevention.

The association between obesity and OC is supported by multiple biologically plausible pathways. Adipose tissue secretes inflammatory cytokines and hormones, which promote tumor cell proliferation and survival. Additionally, obesity‐induced dysregulation of adipokines like leptin and adiponectin disrupts energy metabolism and immune surveillance, creating a microenvironment conducive to oncogenesis [17]. For example, leptin promotes the maintenance of stemness in ovarian cancer cells, whereas low adiponectin levels are associated with reduced apoptosis in tumor cells [18, 19]. These mechanisms align with our observation of higher mortality in obese OC patients.

From 1990 to 2021, the incidence and DALYs attributable to excessive adiposity showed persistent severity and a continuous upward trend. Concurrent with socioeconomic development, excess body weight has become a global health challenge, particularly as an established risk factor for female‐specific malignancies [20]. Our findings indicate that the absolute counts of OC incidence and mortality progressively increased between 1990 and 2021, with projections suggesting this trend will continue at elevated levels over the next 25 years. These observations underscore the urgent need for prioritizing interventions to promote healthier lifestyle practices and reduce the prevalence of metabolic dysfunction.

Existing epidemiological data suggests that individuals with excessive adiposity diagnosed with OC demonstrate disproportionately elevated fatality risks compared to populations within normal weight parameters [21, 22] A longitudinal cohort analysis of 388 female patients with ovarian cancer revealed that progressive elevation in BMI during the peri‐diagnostic period correlated with elevated mortality risks, independent of baseline metabolic status [23]. A population‐based study investigating epidemiological patterns of ovarian cancer within the United States revealed a dose‐dependent association between increasing BMI and disease‐specific fatality rates [24]. While obesity generally worsens cancer prognosis, Bandera et al. reported a counterintuitive survival benefit in advanced‐stage OC patients with elevated BMI [25].

Cancer‐related mortality disproportionately affects geriatric populations and economically disadvantaged regions, with epidemiological surveillance data indicating a marked increase in OC incidence within the 65–84 age cohort. Aligning with these observations, our analysis identifies advanced age (> 65 years) and residence in low SDI areas as critical risk determinants for OC, necessitating prioritized implementation of geriatric‐focused preventive healthcare initiatives and enhanced resource allocation to underserved nations. The immunological surveillance function in ovarian cancer patients exhibits a progressive decline with advancing age. The proportion of patients aged 70 and above receiving complete surgery‐chemotherapy combination therapy is 40% lower compared to younger patients, partially due to concerns regarding treatment tolerance in the frail elderly population [26]. This treatment deprivation phenomenon directly leads to the peak of age‐standardized mortality rate (ASMR) and age‐standardized disease recurrence rate (ASDR) in the older age group. The observed discrepancies in ovarian cancer mortality and incidence between high‐income and low‐income nations may stem from multifactorial determinants, including socioeconomic instability at the community level [27]. The deficient healthcare infrastructure directly contributes to early diagnosis rates for ovarian cancer below 30% in low‐income countries, which are significantly lower than the 78% average in high‐income nations. Furthermore, the North–South disparity in preventive healthcare investments exacerbates the disease burden, with the WHO‐recommended HPV vaccination program achieving 86% coverage in high‐income countries but less than 12% in middle‐ and low‐income nations [28]. Future studies should prioritize elucidating age‐stratified disparities and spatial heterogeneity in the health burden associated with excessive adiposity.

Our findings reveal Europe as the region with the highest obesity‐associated mortality for ovarian cancer in 2021. Europe exhibits high prevalence rates of metabolic syndrome components [29], with 59.5% of adults classified as overweight or obese according to the 2025 World Obesity Atlas. The Western dietary pattern prevalent in many European countries—characterized by high intake of processed foods, refined sugars, and saturated fats—creates a pro‐inflammatory microenvironment conducive to ovarian tumorigenesis [30, 31].

The forecasting framework for OC in our analysis projects a significant escalation in both mortality and DALYs from 2021 to 2046 [32]. Prior epidemiological data corroborate an anticipated surge in obesity‐driven oncological burden over the next two decades. This trajectory may be partially attributed to multifactorial drivers, including demographic transitions, sedentary behavioral patterns, psychosocial stressors contributing to adiposity, occupational carcinogen exposure, and socioeconomic development trajectories. These findings necessitate the implementation of targeted interventions to mitigate the projected trajectory.

This investigation is subject to several methodological constraints. Primarily, the retrospective data repositories lacked detailed documentation of baseline demographic and clinical covariates. Furthermore, the forecasting framework incorporated static assumptions regarding unmodelled confounders across the 25‐year projection window, potentially oversimplifying dynamic risk trajectories. Additionally, the inherent epidemiological characteristics of OC—marked by low population‐level incidence and mortality prevalence—may introduce measurement inaccuracies in GBD‐derived estimates, particularly when applying standardized analytical protocols to rare hematological malignancies.

This study, although addressing partially known confounding through covariate standardization and multiple imputation, could not fully eliminate the possibility of residual confounding. Particularly in assessments of intergenerational effects on long‐term health outcomes, cumulative measurement error and heterogeneity in exposure definitions may further undermine the validity of causal inference. Future research should integrate longitudinal tracking data with molecular epidemiological biomarkers to enhance the precision and external validity of observational causal inference.

## 5 Conclusion

This study advances understanding of metabolic dysregulation's role in ovarian cancer (OC) pathogenesis through three novel epidemiological insights. First, it quantifies the disproportionate impact of elevated BMI on OC mortality in geriatric populations, documenting a 153.2% surge in BMI‐attributable deaths between 1990 and 2021 with peak mortality in adults over 65 years. Second, it reveals stark geographic disparities, demonstrating that low socioeconomic development index (SDI) regions experienced a 3.5–4.1‐fold higher OC mortality increase compared to high SDI areas. Third, predictive modeling using a Bayesian age‐period‐cohort framework projects a sustained 25‐year upward trajectory in OC incidence and disability‐adjusted life years (DALYs), underscoring the urgency of targeted policy action. These findings position metabolic dysfunction as a persistent global health challenge requiring multidimensional interventions.

To address these trends, four evidence‐based policy interventions are prioritized: universal BMI screening for women over 50 years in primary care settings, integrating metabolic health metrics into routine gynecological assessments; targeted deployment of mobile health units and telemedicine networks in low SDI regions to reduce diagnostic delays and improve access to weight management programs; community‐based initiatives combining nutritional education with physical activity promotion, modeled on diabetes prevention programs that reduced metabolic syndrome prevalence by 68.6% in sub‐Saharan Africa; and institutional reforms to prohibit weight‐stigmatizing practices in healthcare delivery while promoting health behavior‐focused frameworks over weight‐centric paradigms. These measures align with successful precedents in obesity‐related cancer prevention and address systemic inequities in OC outcomes.

This investigation acknowledges methodological limitations, including static assumptions in long‐term forecasting models and potential underreporting in low‐incidence regions. Future research should prioritize longitudinal studies exploring adiposity biomarker interactions (e.g., PRL/PRLR pathways) with OC progression to refine risk stratification models. Concurrently, cross‐sector collaborations between health agencies, urban planners, and food industry stakeholders are critical to creating environments that facilitate healthy living at population scale. Such integrated strategies represent a pivotal step toward mitigating the projected acceleration of obesity‐associated ovarian malignancies.

## Author Contributions

Jiwei Li and Dongyong Shan conceived and supervised the work. Xiaolong Li, Zhongyu Liu, and Guangming Fu collected the data; Jiwei Li wrote the manuscript. All named authors meet the International Committee of Medical Journal Editors (ICMJE) criteria for authorship for this manuscript, take responsibility for the integrity of the work as a whole, and have given final approval for the version to be published. All authors read and approved the final manuscript.

## Funding

This study was supported by the Scientific Research Project of Hunan Provincial Health Commission (No. 20231607), the Scientific Research Launch Project for new employees of the Second Xiangya Hospital of Central South University, Beijing Xisike Clinical Oncology Research Foundation (Grant No. Y‐Young2023‐0175), the Natural Science Foundation of Changsha City (CN) (No. kq2403078), and the Natural Science Foundation of Hunan Province (No. 2025JJ60486), National Natural Science Foundation of China (No. 82500253).

## Ethics Statement

The study was conducted in accordance with the Declaration of Hensinki and approved by the appropriate ethics review board of the Second Xiangya Hospital of Central South University, Changsha, China.

## Consent

Written informed consent for publication is not applicable.

## Conflicts of Interest

The authors declare no conflicts of interest.

## Data Availability

The data that support the findings of this study are available from the corresponding author upon reasonable request.
